# Exploring lesbian, gay, bisexual, and queer (LGBQ) people’s experiences with disclosure of sexual identity to primary care physicians: a qualitative study

**DOI:** 10.1186/s12875-015-0389-4

**Published:** 2015-12-09

**Authors:** Marcus Law, Anila Mathai, Paula Veinot, Fiona Webster, Maria Mylopoulos

**Affiliations:** Faculty of Medicine, University of Toronto, Medical Sciences Building, 1 King’s College Circle, Room 3157, Toronto, ON M5S 1A8 Canada; Department of Family & Community Medicine, University of Toronto, 500 University Avenue, 5th Floor, Toronto, ON M5G 1V7 Canada; The Wilson Centre, 200 Elizabeth Street, 1ES-565, Toronto, ON M5G 2C4 Canada

**Keywords:** Sexual identity, Heteronormativity

## Abstract

**Background:**

It has been demonstrated that health disparities between lesbian, gay, bisexual and queer (LGBQ) populations and the general population can be improved by disclosure of sexual identity to a health care provider (HCP). However, heteronormative assumptions (that is, assumptions based on a heterosexual identity and experience) may negatively affect communication between patients and HCPs more than has been recognized. The aim of this study was to understand LGBQ patients’ perceptions of their experiences related to disclosure of sexual identity to their primary care provider (PCP).

**Methods:**

One-on-one semi-structured telephone interviews were conducted, audio-recorded, and transcribed. Participants were self-identified LGBQ adults with experiences of health care by PCPs within the previous five years recruited in Toronto, Canada. A qualitative descriptive analysis was performed using iterative coding and comparing and grouping data into themes.

**Results:**

Findings revealed that disclosure of sexual identity to PCPs was related to three main themes: 1) disclosure of sexual identity by LGBQ patients to a PCP was seen to be as challenging as coming out to others; 2) a solid therapeutic relationship can mitigate the difficulty in disclosure of sexual identity; and, 3) purposeful recognition by PCPs of their personal heteronormative value system is key to establishing a strong therapeutic relationship.

**Conclusion:**

Improving physicians’ recognition of their own heteronormative value system and addressing structural heterosexual hegemony will help to make health care settings more inclusive. This will allow LGBQ patients to feel better understood, willing to disclose, subsequently improving their care and health outcomes.

## Background

Health and health care disparities between lesbian, gay, bisexual, and queer (LGBQ) populations and the general population are well-known [1–4]. LGBQ individuals are at higher risk than heterosexuals for mental health disorders [1, 5]. For example, older men and women in same-sex relationships have greater odds of psychological distress than individuals in married opposite-sex relationships [4], and LGB persons have more depressive symptoms and lower levels of psychological wellbeing than heterosexuals [6]. Some forms of cancers may be more prevalent among the LGBQ population [7, 8] (e.g., anal cancer among HIV-positive men who have sex with men [9]). Sexually transmitted infections are overrepresented, as well, [7, 10], including gay, bisexual, and other men who have sex with men being disproportionately affected by human immunodeficiency virus (HIV) [11]. The LGBQ population has a similarly elevated prevalence of substance use. [5, 7, 12, 13], including tobacco use [14]. LGBQ individuals may also be less likely to engage in preventive health care than their counterparts [2], including screening (e.g., lower rates of Pap tests to screen for cervical cancer in lesbian and bisexual women [15].

Disclosure of sexual identity to a health care provider (HCP) has been linked to health benefits among LGBQ populations [16–18] and their use of health services [19, 20]. Meanwhile, the lack of disclosure to a HCP is associated with health and health care disparities [8, 21] and significantly decreases the likelihood that appropriate health promotion, education and counseling opportunities will be provided [22]. Despite advantages, a significant proportion of the LGBQ population refrains from disclosing sexual identity to HCPs [22–24]. The related sexual and social stigma are linked to the health care inequities that affect this population [2, 25], stressing the importance of holistic strategies to prevention and care.

These findings are particularly important when considering the unique role of the primary care physician (PCP), as compared to other HCPs. Primary care is often the first point of contact in health care [26], and one of the few long-term relationships a patient will have with a physician over his/her lifetime. Moreover, PCPs may treat the families and friends of an LGBQ individual, thus establishing a connection with a group of related persons rather than solely the individual.

PCPs have a role to ensure equitable access to health care for LGBQ patients [27]. Having the opportunity to discuss sexual orientation and gender identity with one’s PCP is an important component of such access. However, surveys have found that most physicians do not ask patients about their sexual orientation [28]. Nonjudgmental discussion and history-taking to elicit information about sexual orientation and gender identity is an essential part of eliminating health care disparities [29] and is part of holistic patient care. The literature suggests that many HCPs assume patients are heterosexual [19, 30, 31]. Heteronormative assumptions and lack of disclosure may lead to suboptimal care [22]. In this study, we sought to understand LGBQ patients’ perceptions of their experiences related to disclosure of sexual identity to their PCP.

## Methods

We used qualitative descriptive methodology for this exploratory work to develop rich, straight descriptions of a phenomenon [32, 33]. Drawing from the tenants of naturalistic inquiry, qualitative descriptive design is a flexible approach that is particularly useful to answer questions relevant to practitioners and is oriented towards producing results that have practical application. Although we used semi-structured interviews with open-ended questions allowing for probes, the interview guide, developed based on expert knowledge, was more structured than those used in other qualitative methods (e.g., grounded theory). The data analysis yielded a description of the data, rather than in-depth conceptual description or development of theory [34].

The study was conducted in a single large urban Canadian city. Our participants were individuals who were 18 years of age or older, fluent in English, self-identified as LGBQ, and had health care provision by PCPs or other HCPs in clinics, emergency rooms, or hospital settings within the previous five years. For the purpose of this study we considered the in-group term “queer’ to include homosexuals [gay, lesbian], bisexuals and pansexuals, reflecting the self-identified characteristics of the interviewees. Following approval by the University of Toronto Research Ethics Board, participants were recruited by advertisement posted at a local community centre. The recruitment poster invited LGBQ individuals to anonymously share their experiences with primary health care by participating in a 30–45 minute interview. Prospective participants contacted the interviewer (AM) directly by email to obtain more information or to express interest in participating in the study. Snowball sampling was also used, whereby participants were asked to suggest potential participants who might supply rich information for the study. Interviews were scheduled at a mutually convenient time and private location. The interviewer (AM) explained the study to each participant and obtained written consent prior to conducting the interview.

**Fig. 1:**
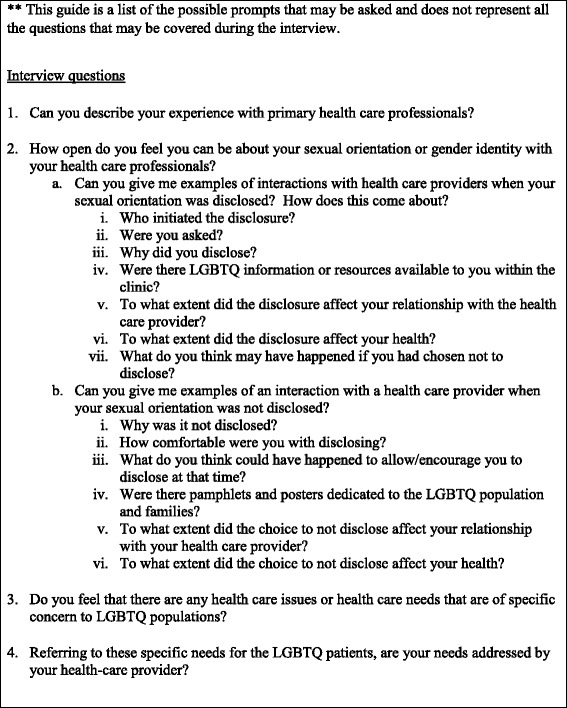
Interview guide

One-on-one in-depth telephone interviews were conducted in 2013 using a semi-structured interview guide (Fig. 1). Interviews were audio recorded, transcribed verbatim, and entered into NVivo qualitative data analysis software (QSR International Pty Ltd; Doncaster, Victoria, Australia) to facilitate analysis. Twelve interviews were conducted to form a rich description of the group of participants at hand, representing a small group of LGBQ patients of a variety of identities. No transgendered or questioning persons came forward to be interviewed. Interviews ranged from 21 to 55 minutes, with most being approximately a half hour in length. Participant characteristics are described in Table 1.

**Table 1:** Participant Characteristics (*n* = 12)

Age (years)	
Mean	32.5
Median	32
Gender identity	
Male/Man	6
Female/Woman	6
Sexual identity/orientation	
Lesbian	1
Gay	6
Bisexual	3
Queer	2
Pansexual	1
* Note: Total does not add to 12 as one participant self-identified as both queer and lesbian	
Highest education	
University (undergraduate, postgraduate)	11
College	1
Employment status	
Employed	9
Unemployed/sabbatical	2
Student	1
In a relationship	
Yes	8
No	4
Age at coming out to anyone (years)	
Mean	19.5
Median	18.5
	*n* = 12

The research team developed an initial coding structure to describe and explore issues about participants’ experiences of disclosure to their PCP, which was revised as analysis continued. Initial codes evolved as data analysis occurred in tandem with the data collection. New data were constantly compared to codes developed from earlier analysis to refine and elaborate the codes and iteratively categorize them into broader themes. During the data collection, we purposively investigated both existing and new codes and identified limitations of the initial coding structure to ensure representativeness of emerging categories. The resulting coding structure was applied to the data set. During final analysis, the research team read the transcripts and identified the thematic structure through iterative relating and grouping of codes.

## Results

Our team identified three main themes related to disclosure of sexual identity to PCPs: 1) disclosure of sexual identity by LGBQ patients to a PCP was seen to be as challenging as coming out to others; 2) a solid therapeutic relationship can mitigate the difficulty in disclosure of sexual identity; and, 3) purposeful recognition by PCPs of the dominant heteronormative value system is key to establishing a strong therapeutic relationship.

First, participants articulated that **disclosure of sexual identity to a PCP is a complex and challenging process equivalent to disclosure to family and friends**. Disclosure to a PCP was part of a broader process of coming out. Being in a clinical rather than social setting alone did not remove the barriers to disclosure.

Patients described having longstanding relationships with their PCPs (e.g., seeing the same physician since they were children), and the participants of this study thought that their PCP assumed all along that they were heterosexual. Disclosing to these PCPs was considered as challenging as coming out to a family member.

“I think that what makes it difficult is, is she’s almost like a, not a mother, but like a family member because I’ve seen her grown up ever since I was a child, so it, the same feeling or pressure of them as your parents kind of applies, I don’t know if that makes sense or not, but that’s a general feeling of why it would be awkward.” (gay male) P12

This difficulty highlighted the burden of disclosing to a PCP and the frustration of having to correct a perceived assumption of a patient’s heterosexuality.

“Well, you know how many times do you have to keep coming up to somebody, you know, if I looked the part, if I dressed like butch-lesbian or something like that, then it would be different I think I just get frustrated, this question I’ve asked myself many times, how many times do you have to come out?” (bisexual female) P5

Participants suggested that the burden and challenge of disclosure could be lessened if physicians asked directly and early in a patient relationship about sexual identity.

“I think simply if they had just asked. If they’d asked and said, you know, made a mention of, you know, if they’d asked about my sexual orientation or if they’d asked, you know, if I had both male and female partners, or transgendered partners, if they had just posed the question, you know, it would have been as easy as that.” (pansexual female) P2

If the PCP did not seek out this information, then participants perceived that it was the responsibility of the LGBQ individual to decide on his/her own whether sexual identity was clinically relevant to the health issues being discussed; thus participants thought that this limited the PCP’s ability to understand and treat the patient as a whole person.

“Not necessarily, I mean, if… you know, had something come up, had I had an issue, I would have talked to her, I would have trusted her. But, as often when it comes to these things, if people don’t bring it up, if I don’t have a reason to bring it up, it doesn’t come up.” (pansexual female) P2

Second, PCPs can leverage a **solid therapeutic relationship to mitigate the difficulty in disclosure of sexual identity**. The relationship is an interactive one, with both the LGBQ patient and the PCP having responsibility and variable influence within the relationship.

Level of anticipated acceptance by PCPs was often judged by participants’ prior clinical encounters in which a PCP’s personality and communication were scrutinized. To ensure disclosure of sexual identity, participants expressed that PCPs need to do more than simply initiate the discussion. From the perspectives of these participants, an effective PCP would build a strong therapeutic relationship and view the patient as a whole person with social context rather than an object with a certain disease. This requires professionalism, compassion, and patient-centeredness on behalf of the PCP, thus facilitating a sense of trust for the patient.

Confidentiality was identified by many as playing an important role in trusting patient-physician relationships. Some participants appeared concerned that the PCP might disclose their sexual identity to their family members, if the physician was treating the participants’ entire family. This brought into question concerns about the PCP’s professionalism and emphasized the nature of family medicine in which the physician treats the whole family unit rather than an individual member.

“[…] some younger people may actually impact them that they need that sort of care but then they don’t feel comfortable coming out, and because they scared that their doctor will share it with their family, I don’t know, it would be something that would be a concern to, the youth” (gay male) P12

Compassion and patient-centredness also seemed to be important characteristics identified by participants. Participants suggested that having the physician convey a sense of understanding the patient in a holistic manner was an important part of a strong therapeutic relationship.

“[…] I feel comfortable with her, we always use up the full time amount, she’ll ask me if there’s anything else, you know, that I’m there for that she can help me with and so I don’t feel rushed. That was the experience I had in the past–feeling not as listened to or a little bit rushed with the doctor. So, yeah, I appreciate that.” (queer/lesbian woman) P1

Professionalism, compassion and patient-centredness seemed to foster trust, which was viewed by participants as a necessary prerequisite for the patient to feel comfortable to reveal his/her sexual identity.

“You know, if I felt like I could have trusted her, then I would have given more information or asked more questions, but, you know, I didn’t trust her to even respect my body, so you know, as it was, so I didn’t really respect, you know, like trust her to respect anything else about me.” (queer female) P4

Third, the **purposeful recognition by PCPs of the dominant heteronormative value system was key to establishing a strong therapeutic relationship**. A therapeutic relationship established through trust, confidentiality and compassion was considered necessary but insufficient to allow some participants to feel comfortable about disclosing their sexual identity. Many participants believed that PCPs additionally need to be deliberate in acknowledging heteronormativity as a social norm in medicine. They provided examples of how they perceived PCPs’ value systems marginalize individuals and how they are complicit if they continue to (knowingly or unknowingly) reinforce a system that people feel judged and marginalized and otherwise excluded.

Communication, as a necessary physician competence, ever present in the patient-PCP relationship, was said to impact the disclosure experience. Language and tone, which conveyed their associated value system, were thought to affect empathy and subsequent comfort with disclosure to a PCP. For example, the use of heteronormative language appeared to negatively influence the perception a patient had of his/her PCP.

“No I don’t think that she’s supportive … just based on language that she uses, and it just seems … no I don’t feel comfortable at all.” (bisexual female) P8

Regardless of whether they could remember experiencing heteronormative language in clinical encounters, participants agreed that gender-neutral language was key to opening discussion about sexual identity. This was perceived to indicate the absence of heteronormative assumptions. The use of heteronormative language seemed to hinder further discussion of a patient’s sexual identity.

“I think that even just removing heteronormative language is a really helpful cue. If somebody asks me if I have a boyfriend, it puts my back up but if somebody asks if I have a partner, that’s a different story and that’s a good indicator that somebody, you know, doesn’t necessarily assume that, you know, my partner is a boy. So that’s one really easy quick way to remove that stigma and open things up.” (queer female) P4

Some thought that the response of the PCP post-disclosure indicated whether the PCP was comfortable with the patient’s sexual identity. A few participants referred to the physician’s tone and the acknowledgement of a patient disclosure of sexual identity. One participant described his physician’s “business as usual” manner after disclosing; the absence of a change in tone led the participant to believe in the physician’s professionalism. Other participants expressed that a lack of acknowledgement seemed to signal that their physician was uncomfortable.

“[…] there wasn’t really much of an acknowledgement or conversation and that in fact, umm, the next, you know, time I went I had wondered if she had actually heard that or–I just felt like, do I have to say it again or, you know, how there wasn’t necessarily any big conversation about it.... but I also didn’t feel like she didn’t answer a question or she was uncomfortable or avoiding or anything. It was just kind of treated like a non-issue but then, yeah, I remember, I think, asking sexual health questions a little bit later on and feeling like, does she still remember that I’m queer? And do I need to say that again and somehow drop it in there–you know, that I don’t have sex with men?” (queer/lesbian woman) P1

When participants perceived the clinical encounter to be framed in a closed fashion, they suggested this led to erroneous heteronormative assumptions on the part of the PCP, thus limiting opportunities for LGBQ patients to disclose their sexual identity.

“I already think that doctors […] they don’t have a lot of time, they just have like 10 minutes for you […] They make a whole lot of assumptions because they don’t have the time.” (bisexual female) P5

## Discussion

Studies over the last decade have shown a significant proportion of the LGBQ population refrains from disclosing sexual identity to HCPs [22–24]. In our study, disclosure of sexual identity by LGBQ patients to a PCP was shown to be as challenging as coming out to families and friends, with participants identifying similar barriers. Participants identified that the power of a strong therapeutic relationship can help mitigate the difficulty in disclosure and included recognition by PCPs of their heteronormative value system.

Our findings highlighted the therapeutic relationship as an interactive relationship, with both the LGBQ patient and the PCP having responsibility and variable influence within the relationship The clinical setting or context is not sufficient to mitigate the barriers of disclosure of one’s sexual identity. Whitehead et al. [35] conducted a contrastive analysis on explicit inclusion or exclusion of “physician as person” in two competency-based frameworks, with a discussion of how the explicit role of the ‘physician as person’ was lost in the CanMEDS Roles. This study suggested that the current dominant model of competency-based education trains future physicians to remove themselves as individuals from the clinical encounter. Use of roles to define physician competencies in outcomes-based educational models has become commonplace [35]. As medical training attends to teaching to such roles, the absence of the “person” role may have implications for how physicians conduct themselves in the clinical encounter. Congruent with Whitehead et al.’s findings, participants in our study viewed their PCPs as part of their social circle and not simply as service providers. Moreover, participants’ highlighted this relationship to be integral to the holistic care of a patient. To ensure the development of therapeutic relationships and reflexive, compassionate, person-centred practitioners, it may be useful to consider how the medical trainee as a person be made visible in the curriculum and in assessment tools [35]. This is made challenging by the imposition of a strong professional identity in medical school that leans toward sameness and homogeneity and ultimately might limit the doctor’s ability to engage as a person in clinical encounters [36].

Also central to our findings was participants’ perceived need for PCPs to treat the person holistically, with attention to social and psychological factors, rather than to just treat the disease. Physicians who were considered by participants to be professional, compassionate and patient-centred embodied the message of the patient as whole, thus fostering a sense of trust in participants. Trust, as a contributor to a strong therapeutic relationship, was thought by participants to promote a healing environment in which the patient felt comfortable to reveal his/her sexual identity to the PCP. St. Pierre [37] similarly highlighted the importance of the patient-provider relationship. Notably, patients who trusted their physician and found communication (one of the six CanMEDS competencies [38]) to be easy were more likely to disclose. Physicians require the skills to develop rapport and trust with patients, and “accurately elicit and synthesize relevant information and perspectives of patients” [39].

Lastly, our data suggest that having PCPs acknowledge their own heteronormative values and how such assumptions may negatively impact the therapeutic relationship would be beneficial to LGBQ patients. Being sensitive to the fact that the LGBQ community remains largely marginalized by a predominantly heteronormative environment is crucial. The challenge is to how best to promote this reflexivity. It is the responsibility of PCPs to ensure that they are cognizant of and explicit about their own social milieus. Our findings also suggest the need for a purposeful recognition by PCPs of their own heteronormative value system to help secure a solid therapeutic relationship. In the role of communicator, ever-present in the PCP-patient relationship, PCPs enable patient-centred therapeutic communication through their language and tone, thus influencing a LGBQ patient to disclose or not. In our study, non-verbal communication impacted the disclosure experience as much as the language chosen. Specifically, participants perceived heteronormative language as an indication of PCPs’ values, which seemed to negatively influence communication, while participants conveyed that gender-neutral language encouraged dialogue about sexual identity. How a PCP responded to a patient’s disclosure of sexual identity through his/her tone or acknowledgement was viewed by participants to signify the physician’s own comfort (or discomfort) with the disclosure. Participants noted heteronormative assumptions in PCPs when the encounter was limited by a restrictive visit (e.g., time constraints prohibiting patient-centred communication) thus limiting opportunities for LGBQ patients to disclose their sexual identity. At the most basic level, medical trainees and physicians should be encouraged to avoid making assumptions regarding patients’ sexual identity. The literature suggests that many HCPs assume, or convey assumptions through questions and behaviour, that patients are heterosexual [19, 30, 31, 40]. If LGBQ persons continued to experience patient-PCP interactions characterized by overt or covert heteronormative communication, then they may feel disenfranchised by the health care system and fail to disclose when advantageous, despite benefits of disclosure. Likewise, verbal and/or non-verbal acknowledgement of a patient sharing his/her identity is important. For example, lack of reaction on the part of a PCP may be erroneously perceived by a patient as a negative response, when in fact the PCP believes no reaction to be an indication of normalizing the disclosure.

Beyond individual PCP values and identity, attention is also needed to the health care system and clinical encounter to support both the PCP and the patient in these discussions. For example, creating supportive environments [8] by having LGBQ-positive signage and clinic materials about various sexual and gender identities and sexual health will help create a more welcoming environment for disclosure and promote ongoing discussions on sexual health. Organizational interventions to allow for more time in clinical encounters [41] and that ensure a place in the electronic health record for such information [28] are possibilities. Employing social justice efforts, adopting relevant policy, and ensuring learning opportunities for current and future staff and physicians to actively engage in reflective and reflexive work are essential to help deflate ever present heterosexual hegemony.

This study has some limitations. Although participants were recruited in Toronto, representing an urban perspective, we do not know where they accessed care or where they were from. This limits ability to make recommendations linked to specific contexts. Also, this study did not interview the participants’ PCPs and, therefore, did not establish how PCPs experienced their LGBQ patient care. However, other research has demonstrated that physicians’ perceptions of patients may be influenced by socio-demographic characteristics [41]. Such perceptions can be deep-rooted and thus difficult to affect change on an individual level. Therefore, as discussed above, applying structural strategies may be more effective.

## Conclusions

Improving physicians’ recognition of their own heteronormative value system and addressing structural heterosexual hegemony will enhance PCPs’ ability to treat the patient as a whole and help to make health care settings more inclusive. This will allow the LGBQ patient to feel better understood as a person and be more willing to disclose, subsequently improving his/her care and health outcomes.

## Availability of Data

For inquiries regarding data supporting the findings presented in this manuscript, please contact the corresponding author, Dr. Marcus Law, Faculty of Medicine, University of Toronto, Medical Sciences Building, 1 King’s College Circle, Room 3157, Toronto, ON M5S 1A8 Canada; Tel: 416-978-4543; Email: marcus.law@utoronto.ca.

## Abbreviations

HCP: health care provider
LGBQ: lesbian, gay, bisexual, and queer
PCP: primary care provider
